# 

**Published:** 1884-03

**Authors:** 


Want of Space.—An extended review of the Report of the Buffalo State Asylum for the Insane is crowded out for want of space. This will be published in the April number.

